# Adipsic Hypernatremia Complicated by Hyperosmolar Hyperglycemic State and Extensive Venous Thromboembolism After Traumatic Brain Injury: A Case Report

**DOI:** 10.1002/ccr3.73832

**Published:** 2026-10-29

**Authors:** Mahshad Ghoddusi, Khashayar Shahverdi, Negar Zareshahi, Zahra Shafizadeh, Mohammad Taghi Talebian, Amir Shokri, Fatemeh Afra

**Affiliations:** ^1^ Department of Nephrology Sina Hospital, Tehran University of Medical Sciences Tehran Iran; ^2^ Department of Anesthesiology and Critical Care Shariati Hospital, Tehran University of Medical Sciences Tehran Iran; ^3^ School of Medicine Tehran University of Medical Sciences Tehran Iran; ^4^ Student Research Committee Tehran University of Medical Sciences Tehran Iran; ^5^ Department of Emergency Medicine Sina Hospital, Tehran University of Medical Sciences Tehran Iran; ^6^ Department of Vascular Surgery, School of Medicine Sina Hospital, Tehran University of Medical Sciences Tehran Iran; ^7^ Department of Clinical Pharmacy Sina Hospital, Tehran University of Medical Sciences Tehran Iran

**Keywords:** adipsia, hypernatremia, hyperosmolar hyperglycemic state (HHS), pulmonary embolism, venous thrombosis

## Abstract

Adipsic hypernatremia is a rare disorder characterized by impaired thirst perception despite severe hyperosmolality, most commonly resulting from hypothalamic dysfunction. Hyperosmolar hyperglycemic state (HHS) may further aggravate dehydration and electrolyte imbalance, increasing the risk of neurologic injury, acute kidney injury (AKI), and thromboembolic complications. Reports describing the coexistence of adipsic hypernatremia, HHS, and extensive venous thromboembolism are limited. A 42‐year‐old man with poorly controlled diabetes and recent traumatic brain injury presented with neurologic decline, severe dehydration without thirst, severe hypernatremia, HHS, and transient renal dysfunction consistent with prerenal injury. Persistent severe hypernatremia despite improvement in hyperglycemia, together with preserved urinary concentrating ability, supported adipsic hypernatremia. Further evaluation revealed extensive deep vein thrombosis (DVT) and bilateral pulmonary embolism (PE), while brain imaging showed only mild diffuse cerebral atrophy. The patient was treated with controlled volume and free‐water replacement, intravenous insulin therapy, anticoagulation, and close monitoring of serum sodium, glucose, renal function, and urine output. Gradual correction of hypernatremia resulted in significant improvement in neurologic manifestations, renal function, and metabolic abnormalities. Following stabilization, he was discharged on apixaban therapy with scheduled oral hydration instructions and outpatient follow‐up. This case highlights a possible synergistic interaction between adipsic hypernatremia and HHS, which may have contributed to severe hyperosmolar stress and extensive thromboembolic complications. Adipsic hypernatremia should be considered in patients with severe hypernatremia and absent thirst sensation, particularly after traumatic brain injury, even when neuroimaging findings are unremarkable.

## Key Clinical Message

Adipsic hypernatremia should be considered in patients with severe hypernatremia and absent thirst after traumatic brain injury, even when neuroimaging appears normal. Concomitant hyperosmolar hyperglycemic state may worsen hyperosmolarity and increase thrombotic risk. Early diagnosis, cautious free‐water replacement, and prompt anticoagulation are critical for favorable outcomes.

## 1 Introduction

Hypernatremia, defined as a serum sodium concentration greater than 145 mEq/L, is a potentially life‐threatening electrolyte disturbance that is frequently associated with significant neurologic and systemic complications, particularly in the setting of severe hyperosmolality. It usually develops because of inadequate water intake, excessive free‐water loss, or impaired thirst regulation [1, 2]. Adipsic hypernatremia is a rare condition in which patients fail to experience thirst despite marked hyperosmolality. This disorder has been described in association with hypothalamic dysfunction, particularly involving the anterior hypothalamic thirst centers, caused by trauma, vascular injury, inflammatory disease, congenital abnormalities, or other central nervous system (CNS) insults [3, 4]. Since thirst plays a crucial role in maintaining osmotic balance, disruption of hypothalamic osmoregulatory pathways may result in profound dehydration and persistent hypernatremia even when renal concentrating function remains intact [4, 5].

Hyperosmolar hyperglycemic state (HHS) is a severe metabolic emergency with high mortality, characterized by profound hyperglycemia, hyperosmolality, and dehydration, most commonly occurring in patients with Type 2 diabetes mellitus [6, 7].

In patients with impaired thirst regulation, HHS may further aggravate free‐water deficit and hypernatremia, potentially obscuring the recognition of underlying adipsia.

The coexistence of HHS can further exacerbate hyperosmolality and intravascular volume depletion, predisposing patients to acute kidney injury (AKI), neurologic impairment, and other serious complications, such as thromboembolic events [8]. In addition to dehydration and hemoconcentration, HHS is associated with endothelial dysfunction, platelet activation, inflammatory cytokine release, and activation of coagulation pathways, all of which contribute to a hypercoagulable state [6, 9, 10]. Nevertheless, reports describing the simultaneous occurrence of adipsic hypernatremia, HHS, and extensive venous thromboembolism remain limited.

Here, we report a patient with poorly controlled diabetes mellitus and recent head trauma who presented with adipsic hypernatremia accompanied by HHS, extensive deep vein thrombosis (DVT), and bilateral pulmonary embolism (PE).

## 2 Case Presentation

A 42‐year‐old man with a history of poorly controlled Type 2 diabetes mellitus, treated irregularly with metformin, and severe head trauma sustained in a motor vehicle accident four months earlier was admitted to the emergency department because of progressive neurologic and functional decline. Approximately two months before admission, his family noted a marked reduction in thirst and spontaneous water intake. Neurologic symptoms gradually progressed, including cognitive slowing, dysarthria, gait instability, generalized weakness, and urinary and fecal incontinence. Progressive unilateral non‐pitting edema of the left lower extremity developed approximately two weeks before presentation.

On admission, the patient appeared acutely ill and clinically dehydrated. He remained conscious but had cognitive slowing, impaired orientation, and dysarthria. Vital signs revealed a blood pressure of 119/88 mmHg, pulse rate of 126 beats/min, respiratory rate of 19 breaths/min, and oxygen saturation of 93% on room air. His height was 185 cm and weight was 84 kg, corresponding to a body mass index of 24.5 kg/m^2^. Physical examination demonstrated non‐pitting edema of the left lower extremity with reduced muscle strength (2/5), primarily attributable to pain. Peripheral pulses were preserved.

## 3 Differential Diagnosis, Investigations, and Treatment

Initial laboratory evaluation demonstrated plasma glucose of 648 mg/dL, HbA1c of 8.0%, and severe hypernatremia (serum sodium 163.7 mEq/L). The calculated effective serum osmolality based on the admission sodium and glucose was approximately 363 mOsm/kg, supporting HHS in the appropriate clinical context. Urine ketones were negative; venous pH was 7.39 and bicarbonate was 22.8 mmol/L. Intravenous insulin therapy and fluid resuscitation were initiated promptly. Additional findings included oliguria, serum creatinine of approximately 1.9 mg/dL, urea of 75 mg/dL, hyperuricemia, and concentrated urine. The transient renal dysfunction was considered predominantly prerenal, resulting from profound intravascular volume depletion. The substantial free‐water deficit was attributed to prolonged impaired thirst and reduced spontaneous water intake, compounded by hyperglycemia‐related osmotic diuresis.

The combination of severe dehydration, persistent hypernatremia, impaired thirst, and preserved urinary concentrating ability raised strong suspicion for adipsic hypernatremia (Table 1 and Figure 1). A concomitant defect in arginine vasopressin secretion was considered; however, clinically significant central diabetes insipidus was unlikely because the patient was not polyuric (urine output approximately 700 mL during the first 24 h) and had a urine osmolality of 1080 mOsm/kg with a specific gravity of 1.036. Formal plasma vasopressin/copeptin measurement and desmopressin challenge testing were not performed; therefore, a subtle partial vasopressin secretory abnormality could not be completely excluded.

**TABLE 1:** The patient's laboratory data on admission.

Parameter	Value	Reference value	Parameter	Value	Reference value
Cr (mg/dL)	1.94	0.7–1.4	CRP (mg/L)	100.9	0–8: Negative
Sodium (mEq/L)	163.7	135–150	Uric acid (mg/dL)	8.3	3.6–8.2
Potassium (mEq/L)	3.9	3.5–5.0	CK (U/L)	822	Up to 195
Calcium (mg/dL)	10.1	8.6–10.3	AST (U/L)	39	Up to 37
Phosphor (mg/dL)	2.3	2.6–4.5	ALT (U/L)	34	Up to 41
HbA1c (%)	8	4.0–6.0	ALP (U/L)	164	120–460
LDH (U/L)	788	< 530	HDL (mg/dL)	42	35–60
CK‐MB (U/L)	27	Up to 24	LDL (mg/dL)	139	< 120
Albumin (g/dL)	4.5	3.5–5.2	TG (mg/dL)	288	Up to 200
WBC (×10^3^/μL)	6.6	4–10.5	Cholesterol (mg/dL)	193	Up to 200
Hgb (g/dL)	12.5	13–17	pH	7.39	7.31–7.41
PLT (×10^3^/μL)	105	150–450	pCO_2_ (mmHg)	37.9	35–59
Urea (mg/dL)	75	18–55	${\mathrm{HCO}}_{3}^{-}$ (mmol/L)	22.8	22–30
SG	1.036	1.008–1.035	Calculated effective Osm (mOsm/kg)	363.4	275–295
Glucose (mg/dL)	648	70–99	Urine ketones	Negative	Negative
Urine osmolality (mOsm/kg)	1080	300–900	aPTT (s)	33	24–35
INR	1.1	1–1.4			

Abbreviations: ALP, alkaline phosphatase; ALT, alanine aminotransferase; aPTT, activated partial thromboplastin time; AST, aspartate aminotransferase; calculated effective Osm, 2 × serum sodium + glucose/18; CK, creatine kinase; CK‐MB, creatine kinase‐MB; Cr, creatinine; CRP, C‐reactive protein; HbA1c, glycated hemoglobin; ${\mathrm{HCO}}_{3}^{-}$, bicarbonate; HDL, high‐density lipoprotein; Hgb, hemoglobin; INR, international normalized ratio; LDH, lactate dehydrogenase; LDL, low‐density lipoprotein; Osm, serum osmolality; pCO_2_, partial pressure of carbon dioxide; PLT, platelets; SG, urine specific gravity; TG, triglycerides; WBC, white blood cells.

**FIGURE 1:**
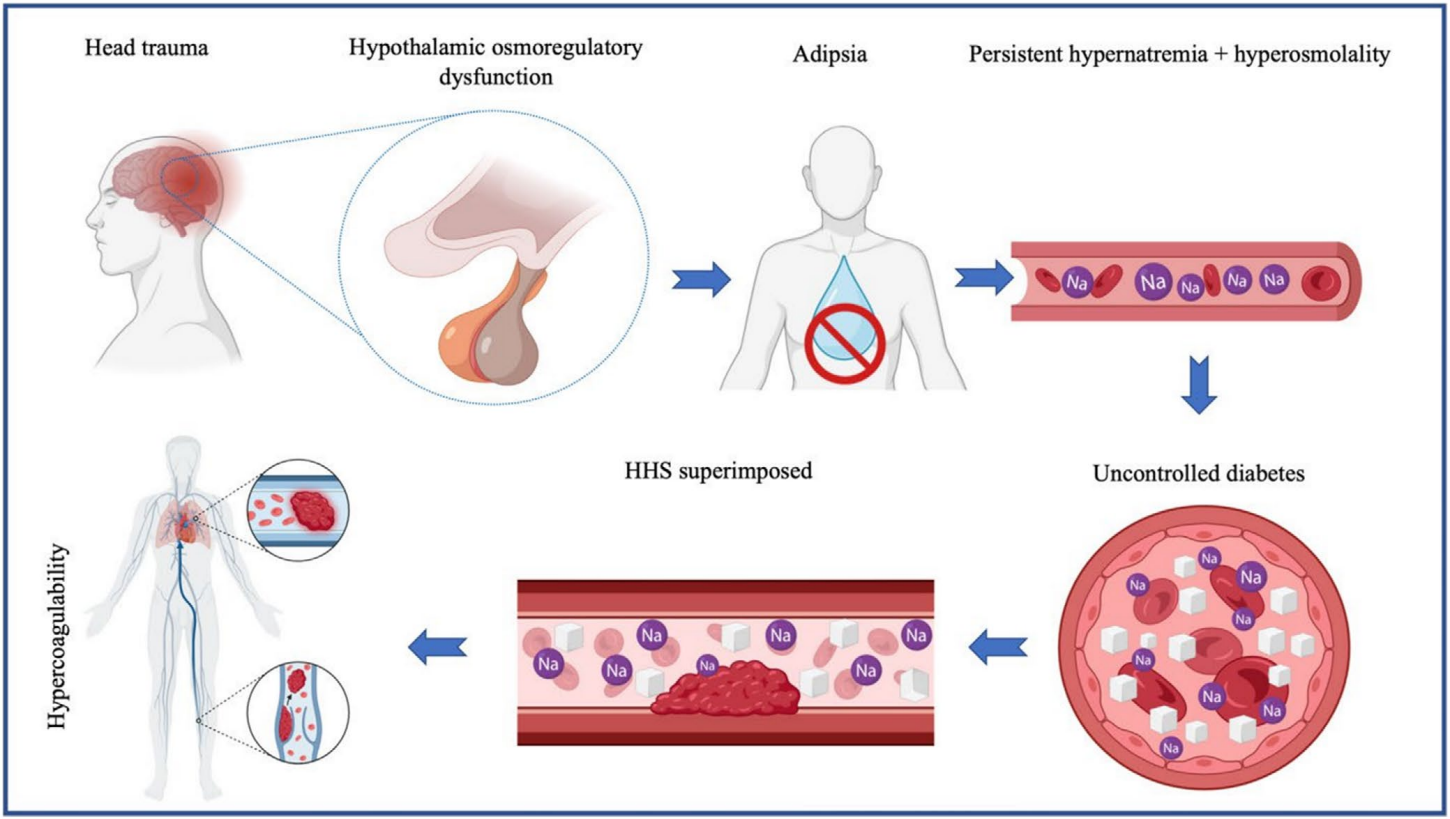
Graphical summary of the case.

Given the approximately two‐month history of impaired thirst, the hypernatremia was considered severe and likely chronic, with a hypovolemic pattern due predominantly to prolonged free‐water deprivation and further aggravated by osmotic diuresis during HHS. Initial management prioritized restoration of intravascular volume before gradual replacement of the free‐water deficit.

Renal ultrasonography demonstrated normal‐sized kidneys (right kidney, 110 mm; left kidney, 103 mm) with preserved parenchymal thickness. A simple 8‐mm cortical cyst was identified in the lower pole of the right kidney. No nephrolithiasis, hydronephrosis, or bladder abnormalities were observed.

Neurologic consultation was obtained to investigate a possible central cause of adipsia. Brain magnetic resonance imaging (MRI), performed on hospital day 5, demonstrated mild diffuse cerebral atrophy without evidence of intracranial hemorrhage, focal gray or white matter lesions, hydrocephalus, mass effect, or midline shift. Magnetic resonance venography (MRV) additionally revealed hypoplasia of the left transverse sinus.

Because of marked unilateral lower‐extremity swelling, Doppler ultrasonography of the left lower limb was performed at admission. Arterial evaluation demonstrated patent common femoral, superficial femoral, popliteal, anterior tibial, and posterior tibial arteries with normal triphasic flow and no evidence of stenosis or plaque formation. In contrast, venous ultrasonography demonstrated non‐compressibility, intraluminal echoes, and absent venous flow within the left external and internal iliac veins, common femoral vein, superficial femoral vein, and popliteal vein, confirming extensive DVT. The inferior vena cava was patent and free of thrombus. These findings indicated that the lower‐extremity manifestations were venous rather than arterial in origin.

Baseline activated partial thromboplastin time (aPTT) was 33 s and INR was 1.1. Unfractionated heparin was not administered. Therapeutic anticoagulation with subcutaneous enoxaparin (80 mg every 12 h) was initiated following neurologic clearance; because low‐molecular‐weight heparin was used, serial PT/aPTT measurements were not used for dose titration. On hospital day 5, worsening hypoxemia and an episode of hypotension prompted computed tomography pulmonary angiography (CTPA), which detected acute bilateral PE involving the right main pulmonary artery and segmental branches of the left lower lobe pulmonary artery. Arterial blood gas analysis revealed severe hypoxemia (oxygen saturation, 66.8%) with mild metabolic acidosis and respiratory compensation. The patient was transferred to the intensive care unit (ICU) for close monitoring; vasopressor support was not required.

Echocardiography demonstrated mild right ventricular enlargement and mildly reduced right ventricular systolic function, consistent with embolic right‐heart strain. Although an episode of hypotension occurred around the time of PE diagnosis, there was no persistent hemodynamic instability and no vasopressor requirement.

Further investigations were performed to evaluate potential contributing systemic conditions. Serologic testing for hepatitis B surface antigen (HBsAg), hepatitis C antibody (HCV Ab), and human immunodeficiency virus (HIV) was negative. Blood cultures remained sterile after 48 h. Nutritional evaluation was unremarkable. Peripheral blood smear (PBS) demonstrated nonspecific erythrocyte morphologic abnormalities without evidence of a definitive hematologic disorder. Further evaluation for thrombophilia and occult malignancy was unremarkable.

Initial intravascular volume resuscitation was performed with 2 L of 0.9% saline, followed by 0.45% saline for controlled correction of the free‐water deficit. After neurologic improvement and once oral intake was considered safe, oral free water was added. Serum sodium decreased from 163.7 mEq/L at admission to 161, 153, 148, and 141 mEq/L on subsequent daily 06:00 measurements and to 136.4 mEq/L at discharge. Plasma glucose decreased from 648 mg/dL at admission to 360, 191, 173, and 126 mg/dL on subsequent daily measurements. HHS resolved on hospital day 3, and the intravenous insulin infusion was discontinued. Clinical neurologic improvement became evident from hospital day 2 and was documented by serial bedside assessment of orientation, communication, gait, and dysarthria; no formal validated cognitive assessment scale was administered. Renal function and hydration status progressively normalized. The overall clinical course is summarized in Figure 2, the potential mechanism of interaction is illustrated in Figure 3, and his laboratory data at discharge are summarized in Table 2.

**FIGURE 2:**
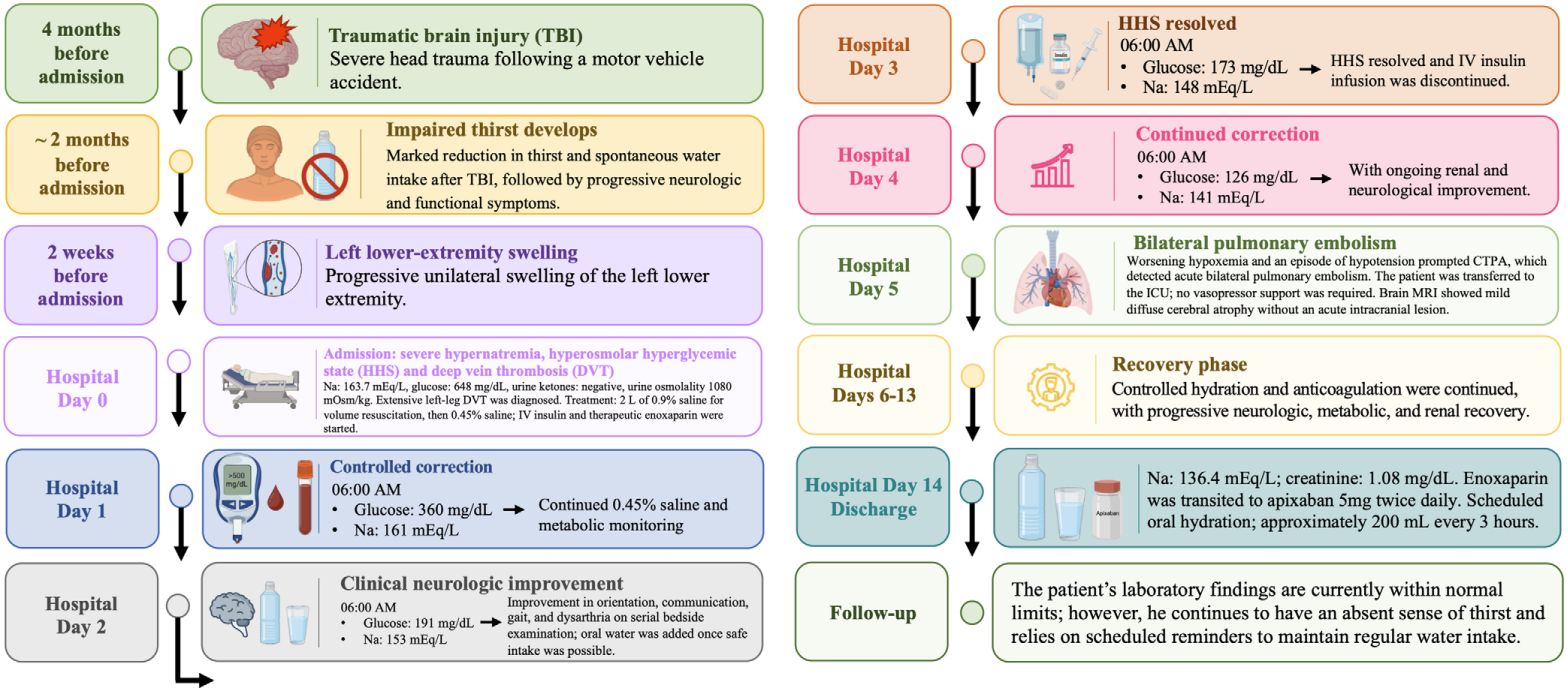
Clinical timeline of presentation, treatment, laboratory correction, and major complications. Daily laboratory values are 06:00 measurements.

**FIGURE 3:**
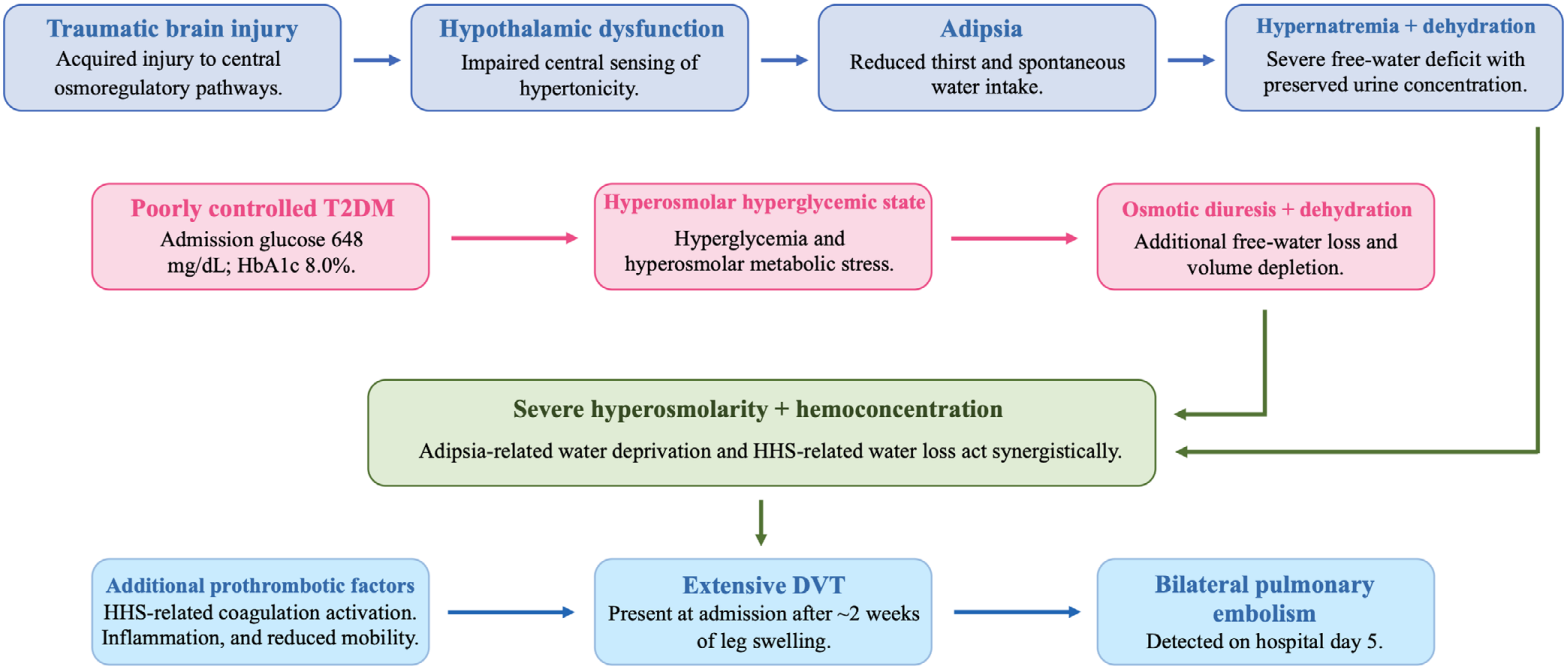
Proposed pathophysiologic interaction among post‐traumatic adipsia, HHS, and venous thromboembolism. TBI‐related impairment of thirst and HHS‐related osmotic water loss likely acted synergistically to produce severe dehydration and hyperosmolarity; HHS‐associated prothrombotic mechanisms, inflammation, and reduced mobility provided additional contributors to venous thromboembolism.

**TABLE 2:** The patient's laboratory data at discharge.

Parameter	Value	Reference value	Parameter	Value	Reference value
Cr (mg/dL)	1.08	0.7–1.4	PLT (×10^3^/μL)	302	150–450
Sodium (mEq/L)	136.4	135–150	Uric acid (mg/dL)	3.4	3.6–8.2
Potassium (mEq/L)	3.6	3.5–5.0	Urea (mg/dL)	22	18–55
WBC (×10^3^/μL)	6.4	4–10.5	CRP (mg/L)	16.5	0–8: Negative
Hgb (g/dL)	11.5	13–17	SG	1.015	1.008–1.035

Abbreviations: Cr, creatinine; CRP, C‐reactive protein; Hgb, hemoglobin; PLT, platelets; SG, urine specific gravity; WBC, white blood cells.

### 3.1 Outcome and Follow‐Up

Following metabolic stabilization, improvement in oxygenation, and continuation of anticoagulation therapy, the patient's overall clinical condition progressively improved.

He was discharged in stable condition on hospital day 14. Enoxaparin was transitioned to apixaban 5 mg twice daily for anticoagulation. He was advised to maintain close outpatient follow‐up for diabetes management and structured hydration because of suspected adipsia, including scheduled oral water intake of approximately 200 mL every 3 h.

## 4 Discussion

In this case, we describe an uncommon but clinically significant presentation of adipsic hypernatremia following traumatic brain injury, complicated by HHS, extensive DVT, and acute bilateral PE. The coexistence of these conditions initially obscured the underlying diagnosis and posed a significant diagnostic challenge.

The complete absence of thirst despite marked dehydration and severe hyperosmolality strongly indicates dysfunction of hypothalamic osmoregulatory pathways. Thirst is primarily mediated by osmoreceptors located within circumventricular organs (CVOs) of the anterior hypothalamus, particularly the organum vasculosum of the lamina terminalis (OVLT) and the subfornical organ (SFO). Based on experimental and clinical studies, the osmotic threshold for the onset of thirst is approximately 294 mOsm/kg [4, 11].

Injury to these structures may disrupt both thirst perception and arginine vasopressin secretion. Although neuroimaging showed no definite hypothalamic abnormality, subtle traumatic injury to these regulatory networks may not be detectable on conventional MRI. Posterior pituitary dysfunction is well recognized after traumatic brain injury, and true adipsic disorders are described as a rare post‐traumatic variant. A similar case of adipsic hypernatremia following traumatic brain injury has been reported despite unremarkable imaging findings [12, 13, 14]. In our patient, formal vasopressin/copeptin measurement was not available; therefore, a subtle partial secretory defect could not be fully excluded.

In this patient, preserved urinary concentrating ability was a key finding, making complete central DI unlikely as the primary cause of hypernatremia. The absence of polyuria, urine output of approximately 700 mL during the first 24 h, urine osmolality of 1080 mOsm/kg, and urine specific gravity of 1.036 indicated effective renal water conservation and supported impaired thirst as the dominant pathophysiologic mechanism. Formal desmopressin challenge testing was not performed. This distinction is clinically important because management strategies differ substantially between adipsic hypernatremia and DI.

The neurologic manifestations, including dysarthria, cognitive dysfunction, gait disturbance, and urinary incontinence, were likely multifactorial. Severe hypernatremia can directly induce neuronal dehydration and cerebral dysfunction [2], while HHS contributes to encephalopathy via hyperosmolar effects [15]. In our patient, the marked neurologic improvement following gradual correction of sodium levels supports a major contribution of osmotic disturbance to the clinical presentation.

A further notable feature of this case was the extensive venous thromboembolism. The thrombotic event was likely multifactorial. Severe dehydration resulting from prolonged adipsia and HHS produced marked hyperosmolarity and hemoconcentration, while HHS itself promotes a prothrombotic milieu through endothelial dysfunction, platelet activation, inflammation, and activation of coagulation pathways [6, 9, 10]. Hypernatremia may contribute to hemoconcentration, thereby potentially increasing blood viscosity and promoting vascular dysfunction [16]. Reduced mobility associated with neurologic decline after traumatic brain injury and systemic inflammation were additional potential contributors. Importantly, extensive DVT was already present at admission after approximately two weeks of unilateral leg swelling, indicating that thrombosis developed before hospitalization. Bilateral PE was detected on hospital day 5 despite ongoing therapeutic anticoagulation and was considered part of the same extensive thromboembolic process rather than proof of a new in‐hospital event. Evaluation for thrombophilia and occult malignancy was unrevealing. Right ventricular dilation on echocardiography reflected pulmonary vascular strain; an episode of hypotension occurred around the time of PE diagnosis, but there was no persistent hemodynamic instability and no vasopressor requirement.

This case highlights the synergistic interaction between adipsic hypernatremia and HHS, which together intensified hyperosmolar stress and contributed to extensive thromboembolic complications. The differential diagnosis included osmotic diuresis due to uncontrolled diabetes, DI, inadequate water intake, and pseudohypernatremia; however, persistent severe hypernatremia despite improvement in hyperglycemia, preserved urinary concentrating ability, and absence of thirst supported the diagnosis of adipsic hypernatremia. Although neuroimaging was unremarkable, prior traumatic brain injury remained the most likely precipitating factor given the temporal association with symptom onset. This case also emphasizes the importance of gradual correction of chronic hypernatremia, as rapid sodium reduction may precipitate cerebral edema and neurologic deterioration. Careful free‐water replacement with close laboratory monitoring resulted in favorable neurologic and renal recovery, underscoring the reversibility of osmotic derangements when promptly recognized and appropriately treated.

From a clinical perspective, management requires a multidisciplinary approach focused on controlled sodium correction, structured fluid replacement, close metabolic monitoring, and early consideration of thromboembolic prophylaxis in patients with concurrent hyperosmolar and neurologic risk factors. Long‐term prevention should emphasize patient education and adherence to scheduled water intake to reduce recurrence.

Given the limited literature describing the coexistence of adipsic hypernatremia, HHS, and extensive venous thromboembolism [17], this report further supports the association between severe hyperosmolar states and clinically significant thrombotic complications requiring vigilant monitoring and timely anticoagulation.

## 5 Conclusion

Adipsic hypernatremia should be considered in patients presenting with severe hypernatremia and absent thirst, particularly following traumatic brain injury. Concomitant HHS may exacerbate dehydration, neurologic dysfunction, and thrombotic risk. This case demonstrates that profound hyperosmolar states can precipitate extensive venous thromboembolism and PE even in relatively young patients. Early recognition, careful correction of sodium abnormalities, controlled fluid resuscitation, and prompt anticoagulation are essential to optimize outcomes and prevent potentially fatal complications.

## Author Contributions

**Mahshad Ghoddusi:** conceptualization, investigation, data curation, project administration, supervision, writing – review and editing, methodology. **Khashayar Shahverdi:** conceptualization, investigation, formal analysis, methodology, writing – original draft, writing – review and editing. **Negar Zareshahi:** data curation, investigation, resources. **Zahra Shafizadeh:** conceptualization, data curation, investigation, resources, validation. **Mohammad Taghi Talebian:** writing – review and editing. **Amir Shokri:** writing – review and editing. **Fatemeh Afra:** conceptualization, investigation, validation, formal analysis, methodology, writing – original draft, writing – review and editing.

## Funding

The authors have nothing to report.

## Ethics Statement

This case report was conducted in accordance with the ethical principles of the Declaration of Helsinki.

## Consent

Written informed consent for the publication of this case report, including all accompanying laboratory data, was obtained from the patient, who agreed to the disclosure of the relevant medical information.

## Conflicts of Interest

The authors declare no conflicts of interest.

## Data Availability

The data that support the findings of this study are available from the corresponding author upon reasonable request.
